# The ripple pond: enabling spiking networks to see

**DOI:** 10.3389/fnins.2013.00212

**Published:** 2013-11-15

**Authors:** Saeed Afshar, Gregory K. Cohen, Runchun M. Wang, André Van Schaik, Jonathan Tapson, Torsten Lehmann, Tara J. Hamilton

**Affiliations:** ^1^Bioelectronics and Neurosciences, The MARCS Institute, University of Western SydneyPenrith, NSW, Australia; ^2^School of Electrical Engineering and Telecommunications, The University of New South WalesSydney, NSW, Australia

**Keywords:** object recognition, spiking neural network, neuromorphic engineering, image transformation invariance, view invariance, polychronous network

## Abstract

We present the biologically inspired *Ripple Pond Network* (RPN), a simply connected spiking neural network which performs a transformation converting two dimensional images to one dimensional temporal patterns (TP) suitable for recognition by temporal coding learning and memory networks. The RPN has been developed as a hardware solution linking previously implemented neuromorphic vision and memory structures such as frameless vision sensors and neuromorphic temporal coding spiking neural networks. Working together such systems are potentially capable of delivering end-to-end high-speed, low-power and low-resolution recognition for mobile and autonomous applications where slow, highly sophisticated and power hungry signal processing solutions are ineffective. Key aspects in the proposed approach include utilizing the spatial properties of physically embedded neural networks and propagating waves of activity therein for information processing, using dimensional collapse of imagery information into amenable TP and the use of asynchronous frames for information binding.

## Introduction

**Figure 1:**
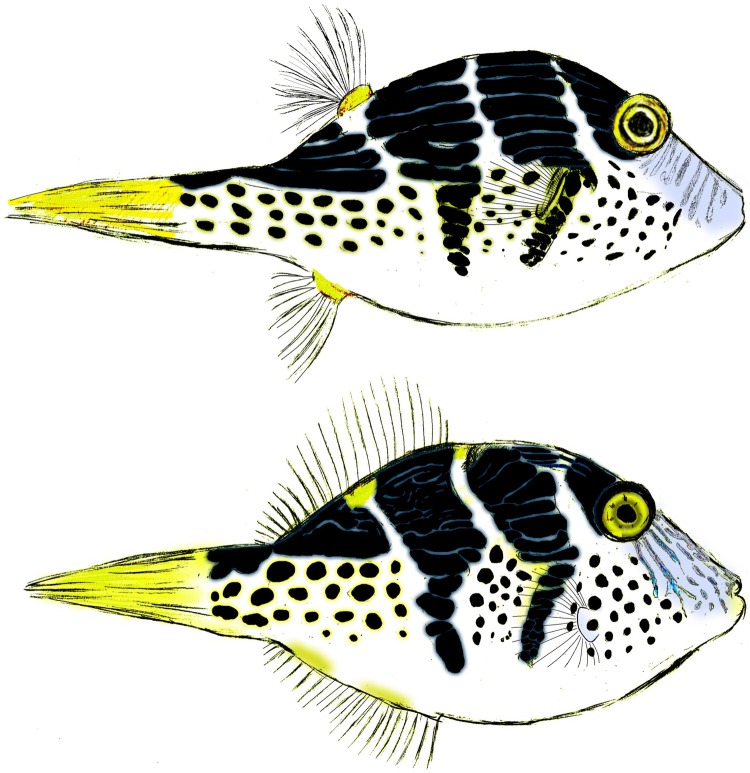
**Batesian mimicry: The highly poisonous pufferfish, *Canthigaster valentine* (top) and its edible mimic *Paraluteres prionurus* (bottom)**. The remarkable degree of precision in the deception reveals the sophisticated recognition capabilities of the neural networks of local predatory reef fish (Caley and Schluter, 2003). These networks despite being orders of magnitude smaller than those of primates seem capable of matching human vision in performance and motivate the investigation of very simple solutions to the problem of visual recognition. (Note: the dorsal, anal and pectoral fins are virtually invisible in the animals' natural environment).

How did the earliest predators achieve the ability to recognize their prey regardless of their relative position and orientation? What minimal neural networks could possibly achieve the task of real-time view invariant recognition, which is so ubiquitous in animals, even those with miniscule nervous systems (Van der Velden et al., 2008; Avargués-Weber et al., 2010; Tricarico et al., 2011; Gherardi et al., 2012; Neri, 2012), as evidenced by mimetic species (Figure 1). yet so difficult for artificial systems? (Pinto et al., 2008). If realized such a minimal solution would be ideal for today's autonomous imaging sensor networks, wireless phones, and other embedded vision systems which are coming up against the same constraints of limited size, power and real-time operation as the earliest sighted predators.

In this paper we present an element of such a simple network. We call this subsystem the Ripple Pond Network (RPN). The name Ripple Pond alludes to the network's fluid-like rippling operation. The RPN is a feed-forward time-delay spiking neural network with static unidirectional connectivity. The network is responsible for the transform of centered input images received from an upstream salience detector into spatio-temporal spike patterns that can be learnt and recognized by a downstream temporal coding memory network.

### The view invariance problem

In the 2005 book, “23 Problems in Systems Neuroscience” (van Hemmen and Sejnowski, 2005), the 16th problem in systems neuroscience, as posed by Laurenz Wiskott, is the view invariance problem. The problem arises from the fact that any real world 3D object can produce an infinite set of possible projections on to the 2D retina. Leaving aside factors such as partial images, occlusions, shadows, and lighting variations, the problem comes down to the shift, rotation, scale, and skew variations. How then, with no central control could a network like the brain learn, store, classify and recognize in real time the multitude of relevant objects in its environment?

Generally most biologically based object recognition solutions have been based on vertebrate vision, in particular mammalian vision, and have used either statistical methods (Sountsov et al., 2011; Gong et al., 2012), signal processing techniques (such as log-polar filters) (Cavanagh, 1978; Reitboeck and Altmann, 1984), artificial neural networks (i.e., non-spiking neural networks) (Nakamura et al., 2002; Norouzi et al., 2009; Iftekharuddin, 2011), and more recently, spiking neural networks (Serre et al., 2005; Rasche, 2007; Serrano-Gotarredona et al., 2009; Meng et al., 2011).

Since many of the approaches above are based on mammalian vision and strive to achieve the accuracy and resolution of mammalian vision, they are very complex and can only be truly implemented on computers (Nakamura et al., 2002; Serre et al., 2005; Jhuang et al., 2007; Norouzi et al., 2009; Iftekharuddin, 2011; Meng et al., 2011; Gong et al., 2012), sometimes with very slow computation times. Other implementations that have been demonstrated on hardware (Rasche, 2007; Serrano-Gotarredona et al., 2009; Folowosele et al., 2011) have been successful in proving that vision can be achieved for small, low-power robots, UAVs, and remote sensing applications.

The few models of invertebrate visual recognition have had an explanatory focus (Horridge, 2009; Huerta and Nowotny, 2009) and, not being developed for the purposes of hardware implementability, assume highly connected networks not suitable for hardware.

CMOS implementations of bio-inspired radial image sensors are most closely related to the RPN however when it comes to recognition, these sensors ultimately interface with conventional processors (Pardo et al., 1998; Traver and Bernardino, 2010) rather than spiking neural networks as is the case with the RPN.

### Beginning at the end: 2D rate coding memory vs. 1D temporal coding memory

In all the previously listed approaches to the view invariance problem the learning and memory system of network responsible for recognition has had a 2D structure. This approach is intuitive. A memory system that matches the input channel (the retina) in dimension makes sense from an engineering perspective. A 2D signal should interface with a 2D memory system. However, from a biological perspective there is no evidence for the existence of such a 2D or grid structured memory system in any organism. Furthermore and more critically from a computational perspective, the standard 2D memory approach necessitates the precise alignment of perceived objects to a stored canonical 2D template in terms of their scale, position and angle, a computationally centralized and biologically implausible operation. The mechanisms by which this matching of a 2D image to a 2D template is accomplished makes up a significant portion of the field of machine vision. This 2D model of visual memory sits in contrast to the more general model of memory as used by computational neuroscientists not focused on vision, and in particular those specializing in memory systems.

Among the later group, temporal coding networks have been proposed in the last two decades as biologically plausible and computationally useful models (Jaeger, 2001; Maass et al., 2002; Izhikevich, 2006; Tapson et al., 2013). A temporal coding memory network is a type of spiking neural network which uses spatio-temporal patterns of spikes to represent information asynchronously whereas classic artificial neural networks discard timing information by modeling neuron firing rates sampled synchronously by a central clock. This additional use of asynchronous temporal information results in greater energy efficiency and speed (Levy and Baxter, 1996; Van Rullen and Thorpe, 2001) motivating realization of the model in hardware (Wang, 2010; Wang et al., 2011; Hussain et al., 2012).

In this model a neuron can be seen as a memory unit which learns and stores via its dendritic weights and delays a particular spatio-temporal pattern.

**Figure 2:**
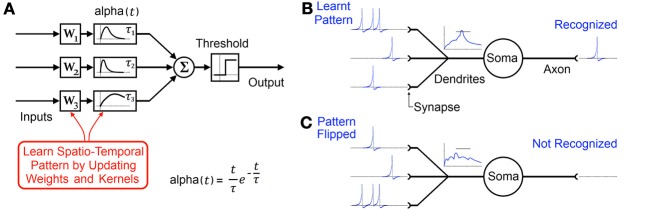
**(A)** Typical model of a single element in a distributed temporal coding memory network with synaptic alpha functions used as decaying synaptic kernels producing a decaying memory of recent spikes. **(B)** Biological representation of the same element. Through adaptation of synaptic weights and kernels a specific spatio-temporal pattern is learnt by the neuron. **(C)** Flipping the pattern as would happen if a 2D image were rotated by 180 degrees results in the pattern not being recognized.

This temporal coding memory model is a content addressable, distributed network comprising of many spiking neurons connected to each other via multiple pathways. The network, through dynamic adaptation of synaptic weights (W_1_, W_2_, W_3_ in Figure 2) and decaying synaptic kernels such as alpha functions with time constants (τ _1_ τ _2_ τ_3_ in Figure 2), makes particular neurons exclusively responsible for particular inter-spiking intervals. It achieves this by continuously adapting its parameters to maximize recognition at its output in response to the statistics of its input. A longer spatio-temporal pattern can be stored in such a network of neurons by the addition of cascading neurons and in turn learning these weights and time constants (Paugam-Moisy et al., 2008; Ranhel, 2012). Subsequently the network's “output” can be measured as the relative activation of certain neurons which individually or in concert indicate the recognition of a certain pattern.

However just as with rate coding models, temporal coding memory systems tasked with recognition also cannot directly interface with the retina since they also expect their learnt patterns to appear via the same channels every time (see Figure 2).

Motivated by and working backwards from this time-based model of memory we propose the RPN system which instead of attempting to align a 2D image to a 2D template, converts the 2D image to a 1D Temporal Pattern (TP) suitable for temporal coding memory networks. We then extend the system by using multiple RPNs in parallel each sensitive to particular features. These parallel RPNs convert the 2D image to an M-dimensional spatio-temporal pattern. Thus the proposed RPN system can be viewed as a simple neuronal transformation which connects the 2D retinotopically mapped inputs to a biologically plausible temporal coding memory model.

## Materials and methods

### The RPN network

A central aim of the hardware oriented RPN approach is to obtain the most functionality from a minimally connected network. The limiting factor of connectivity, though far less significant in biology, frequently constrains hardware implementations and yet is not often considered in the development of artificial neural network algorithms. Approaches which consider such limitations at the designs stage facilitate efficient hardware implementation (Sivilotti, 1991; Hall et al., 2004; Furber et al., 2012).

### An upstream shift invariant salience network

**Figure 3:**
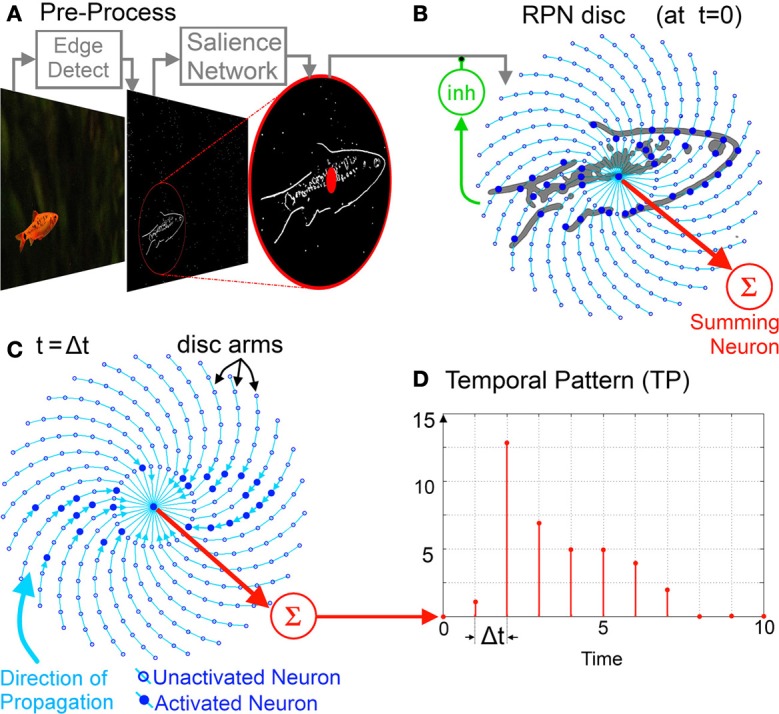
**The spiral RPN System Diagram: raw image to TP. (A)** Pre-processing stage, from the raw image feature(s) extracted and input to a salience network which detects the most salient region and translates it to the RPN aperture. **(B)** The image is then projected on to the RPN disc with the RPN taking in one frame at a time via its inhibitory feedback neuron (inh). **(C)** The projected image is then processed via its unidirectional inwardly pointing disc arms. The image collapses inward toward the center along the arms where it is summed by the Summing Neuron. **(D)** The output of the summing neuron is an integer valued temporal pattern which can be processed by memory. For visual clarity the disc illustrated comprises of only of thirty arms (Φ = 30) and ten neurons per each arm *n* = 0 … 9 with *n* = 0 being the central neuron.

As shown in Figure 3, the RPN receives an image as the spatio-temporal, high-pass filtered activation pattern of neurons on a conceptual 2D sheet representing the field of attention that has been produced by an upstream salience detection system. By using a sliding window of attention and focusing it onto a single salient object at a time the salience detector effectively allows the overall network to operate in a shift invariant manner. The field of computational and biologically-based salience detection is extensive with a wide range of models, techniques, and approaches (Itti and Koch, 2001; Vogelstein et al., 2007; Gao and Vasconcelos, 2009; Drazen et al., 2011). The proposed RPN system does not require any specific salience model having a centered input image as its only requirement. For simplicity, however, we may assume the upstream salience network to consist of only a motion detector, which physically fixes the creature's gaze onto a moving object. In fact, this simplified system is not far off the mark in the case of many organisms (Dill et al., 1993; Land, 1999) and may serve in robotics applications where energy and hardware are also limiting factors.

### Input images ripple inwards on the RPN disc

After centering by the salience network, the incoming image stimulates the neurons distributed on a disc. The disc consists of Φ arms and N neurons per arm as shown in Figure 3.

Functionally these neurons represent simple binary relays with fixed unit delays between them. More complex neuron models could also be used but this would incur an increased hardware cost (Indiveri et al., 2011). The neurons have one outbound connection to the next neuron on their arm. This connectivity is unidirectional pointing inwards toward the center of the disc and each neuron *a*_*n*−1, φ_ can be activated at time (t) if its upstream neuron (on the same spiral arm but further away from the disc center), *a*_*n*, φ_ transmits a pulse to its input at (*t* – –Δ*t*). Thus, starting from the disc edge (*n* = *N* − 1), the neuronal connections radiate inward to the central neurons on the disc (*n* = 1). This inward connectivity creates a rippling effect and since the neurons act as relays with a small delay, stimulation of a few neurons at the edge of the disc produces a small wave of activation which travels inward along the disc arms stimulating succeeding neurons in turn and ending at the disc center [see equation (1)].

For *n* = 1 … *N* − 1 For φ = 1 … Φ

(1) $$a_{(n-1),\varphi }(t)=a_{n,\varphi }(t-\Delta t)$$

where *a*_*n*, φ_ (*t*) is the activity on the *n*th neuron on arm φ at time *t*.

### A summing neuron outputs a temporal pattern

The inner most ring of neurons (*n* = 1) as well the single central neuron (*n* = 0) all connect to a common Summing Neuron (red sigma in Figure 3) that outputs an integer-valued TP that can be represented as an *N* element vector [Equation (2)].

(2) $$TP_{sum}\left(t\right)=\sum_{\varphi =1}^{\Phi }a_{0,\varphi }\left(t-\Delta t\right)+a_{1,\varphi }\left(t-2\Delta t\right)$$

where:

*TP*_*sum*_(*t*) is the TP output of the summing neuron,

Φ is the total number of neurons on the disc,

*a*_1, φ_ (*t*) is the activity of the inner most ring of neurons (*n* = 1) on arm φ at time *t*.

*a*_0, φ_ (*t*) is the activity of the central neuron (*n* = 0).

The summing neuron sums the activity of the disc's central neurons (*n* = 1 and *n* = 0). Where the activity of the RPN neurons are digital, as is the case here, the summing neuron outputs an integer valued spike at every time step generating the TP that is the RPN's output. From the geometry of the disc it is clear that this output TP is rotationally invariant. More subtly, as the neuron distribution on the disc is uniform, the TP resulting from a scaled object is a rescaled (in time and in magnitude) version of the TP produced from the original object. This dimensional collapse of the object's rotational and scale variance into a TP greatly simplifies the task of recognition by downstream memory systems.

### An inhibitory neuron acts as an asynchronous shutter

Recalling that in addition to receiving inputs from their outer neighbor, all neurons are also sensitive to an incoming image. Here the neurons function as radially distributed, inwardly connected pixels on a circular retina such that any pixel/neuron could be activated either via its outer neighbor or from its corresponding sensor. This double activation path means that if there is continuous input from the incoming image, say via an asynchronous frameless vision sensor or an actual biological retina, the information carried on the disc during the processing phase will be corrupted and the generation of unique TPs made impossible.

**Figure 4:**
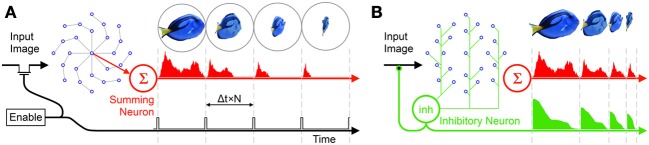
**Two frame generation approaches: (A) A periodic enable signal projects new frames on to the RPN, (B) the inhibitive neuron is connected to all neurons on the disc**. As the disc activation collapses inward along the arms and leaves the disc via the summing neuron, the total activation reaching the inhibitive neuron also falls. Once the disc activation reaches zero, the path of the input image is unblocked allowing the next frame to be projected. As the target object moves away and the incident image becomes smaller it takes less time for the activation to clear the disc disabling the inhibitory neuron sooner and projecting the frame. In this way the RPN frame rate varies dynamically to maximize TP generation. Note that in the RPN disc shown, Φ = 8 (arms) and *N* = 4 (neurons per arm).

In order to prevent the corruption of the RPN's rippling operation by new input images some form of shuttering is required. One way to control the projection of new image frames onto the disc is via a periodic enable signal which enables image projection at Δ*t* × *N* time intervals ensuring that the activation due to the last frame has cleared the disc shown in Figure 4A. The drawback of this approach, however, is that if the projected object size is smaller than the disc (which is almost always the case), significant time is wasted in processing the empty outer regions of the disc, during which time new incoming information could potentially be lost.

A more efficient approach is the use of an asynchronous shutter. To this end, in addition to being sensitive to an incoming image and synapsing onto their inner neighbors along the disc arm, all neurons on the disc also connect via a third path to an inhibitory neuron (green neuron labeled *inh* in Figure 4B) such that the inhibitory neuron carries information about the net activity of *all* neurons on the disc. In a hardware context this signal may simply correspond to the net power consumption of the disc.

(3) $$Inh\left(t\right)=\sum_{\varphi =1}^{\Phi }\sum_{n=0}^{N-1}a_{n,\varphi }\left(t\right)$$

where:

*Inh*(*t*) is the output of the inhibitory neuron.

The output of the inhibitory neuron feeds back inhibitively onto the visual pathway carrying the image to the disc. The Inhibitory neuron blocks this pathway preventing further transmission of the image. In this way the neuron ensures that as long as any activity remains on the disc (i.e., while RPN is processing the image) no new image will be projected onto it, see Figure 4B. The drawback of both these solutions is that their sharp sampling operations are not biologically plausible. A more nuanced solution involves the use of laterally inhibitive pathways to bunch neural signals into frame-like wavefronts (Brunel, 2000; Hamilton and Tapson, 2011; Afshar et al., 2012; McDonnell et al., 2012) we consider this solution in the later discussion section.

### Neuron placement algorithm: uniform distribution vs. log-polar/space variant distributions

It may be apparent by inspection that the RPN is invariant to rotation due to the radial symmetry of the disc in a fashion similar to log-polar sensor placement schemes. However, in contrast to log-polar and other space variant schemes, the density of neurons on the RPN disc remains approximately uniform as we move away from the center. This symmetric but uniform distribution was achieved by placing the *n*th neuron on each arm at a distance $r_{n}=\sqrt{n}$ from the center and spiraling the arms at each step n by an offset angle β_*n*_. A search algorithm was used to determine β_*n*_ for each new ring of neurons. Random angular offsets were tried (1000 trials/*n*) and the minimum distance to previously placed neurons calculated. The trial with the largest minimum distance was selected for each *n*. The resulting neuron distributions from this randomized algorithm display, highly structured spiral forms.

**Figure 5:**
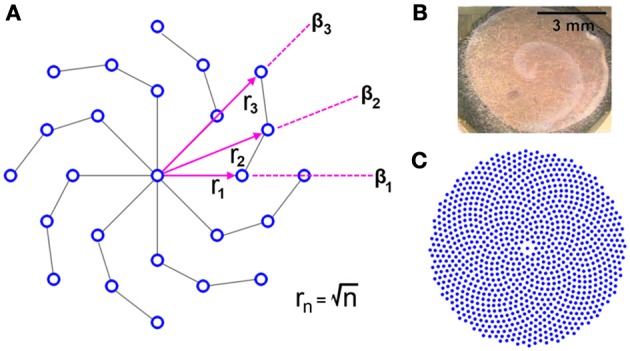
**(A)** Generating uniform global and local neuron density in a radially symmetric distribution via an adaptive algorithm that varies the angular of new neurons β _n_ such distance to the nearest neighbor is maximized results in a spiral structured disc. The disc is shown with (Φ = 8, *N* = 4). **(B)** Spiral propagating waves of neural activity on the chicken retina due to excitation. Image from (Yu et al., 2012). **(C)** The spiral structure at larger scales RPN disc with (Φ = 8, *N* = 128).

Such spiral structural symmetry as well as the spreading of wave-like activation has been observed in the visual pathways of a range of animals from the retina to the higher layers of the visual cortex (see Figure 5B) suggesting possible utility in visual processing (Dahlem and Müller, 1997; Huang et al., 2004; Wu et al., 2008; Dahlem and Hadjikhani, 2009). In the context of artificial systems the use of wave-like dynamics for computation and recognition has only recently begun (Adamatzky et al., 2002; Fernando and Sojakka, 2003; Maass, 2007; Izhikevich and Hoppensteadt, 2009).

In contrast log-polar approaches to vision have developed over several decades (Cavanagh, 1978; Reitboeck and Altmann, 1984). Yet these approaches have had the critical flaw of being particularly sensitive to centering, a problem demonstratively absent in biology. The problem with the log-polar solution is it represents a local minima in the solution space. Its space variant distribution provides a useful automatic scale invariance but critically closes the path to extension with respect to translation invariance since the non-uniform distribution cements the non-uniform behavior of the system in response to a translated image. Furthermore in the context of biological plausibility, the central assumption used by advocates, that the retino-cortical mapping of the mammalian visual system represents a mathematical log-polar transformation (Traver and Bernardino, 2010), is subject to controversy in the neuroscience community (van Hemmen and Sejnowski, 2005), as it fails to explain both off-center recognition or the fact that the fovea, which represents the central 2° of the visual field and is primarily responsible for object recognition, has a uniform retino-cortical mapping (Gattass et al., 2005).

The use of a uniform distribution on the other hand not only represents a more efficient use of available sensor/neuron space (a critical factor both in hardware and in biology), and a more accurate representation of the part of the visual system actually responsible for recognition, but most importantly keeps open the path toward a general solution that is invariant to all sources of variance including translation.

### Time warp invariance in memory enabling scale invariance in vision

**Figure 6:**
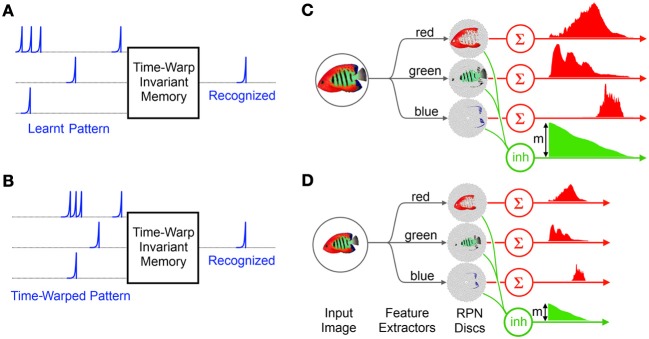
**Time Warp invariant memory network and the RPN's scale invariance: (A) the memory network learns a particular spatiotemporal pattern (B) The memory network recognizes a time warped version of the learnt pattern (C) The RPN system generates a spatio-temporal pattern from the projected image of reef fish via simple color based feature extractors**. The magnitude *m* of the initial activation of the inhibitive neuron carries total activity due to the image greatly simplifying the normalization operation of the memory network. **(D)** A smaller version of the image takes less time to collapse and leave the disc generating a time warped version of the original TP.

One of the important capabilities of temporal coding memory systems is the recognition of the same pattern when presented at different speeds and magnitudes (Kohonen, 1982; Paugam-Moisy et al., 2008; Gütig and Sompolinsky, 2009; Carandini and Heeger, 2012; Tapson and van Schaik, 2013). Such systems can use temporal cues embedded in the input signal or a separate signal carrying normalization information to modify the internal parameters of their dynamic systems such as the time constants of synaptic kernels, to slow down or speed up the system to the signal calibrating their speed of operation to achieve invariance to signal speed, Figures 6A,B. This scheme, often described as *shunting inhibition* (Koch et al., 1983; Volman et al., 2010), is a fundamental element in neuro-computation present in many neural systems and responsible for tasks such as enhancement of signal to noise ratio, control of signal propagation speeds and control of the dynamic range of neural signals (Wills, 2004; Carandini and Heeger, 2012).

One of the consequences of the uniform distribution of neurons on the RPN disc is that rescaled input images produce TPs which are rescaled temporally and in magnitude as shown in Figures 6C,D. This is because relative to a larger image a smaller image activates fewer numbers of neurons on the disc and the neurons activated are correspondingly closer to the center than for a larger image and so the wavefront of activity arrives sooner at the summing neuron than for the larger image. Thus the resulting TP is a time and magnitude scaled version of the original which can be recognized by a time-warp invariant temporal coding memory network.

In this context the RPN approach is particularly useful since it provides a ready normalization signal via the initial activation level of the inhibitory neuron, *m* in Figures 6C,D, which corresponds to the initial activation of every neuron on the RPN discs. The rising edge of the inhibitory neuron not only signals the exact start time of the TPs but its initial magnitude *m* representing the size of the image can be used by the time-warp memory to achieve normalization via an inverse relationship to the memory system's time constant (Gütig and Sompolinsky, 2009). Furthermore since the inhibitory signal arrives immediately after image projection it can be sent directly to memory *before* the first segments of the TPs arrive.

It is worth noting that in contrast to the uniform distribution scheme described, an RPN disc with a log-polar neuron distribution would, given an image at two different scales, generate the same TP, enabling the system to interface with temporal coding memory networks that are not time-warp invariant. This advantage however, as detailed earlier, is outweighed by the unsuitability of the log-polar scheme for extension to a more general translation invariant solution.

### Multiple parallel heterogeneous discs result in higher specificity

A drawback of the collapse of a feature rich 2D image into a TP is that information can be lost. To counteract this loss of information, the simple RPN system can be extended such that instead of using a single disc, the input image can be projected onto multiple discs operating in parallel each of which extracts different feature maps from the raw image. The simplest features can be extracted at the sensor level these include color, motion, and intensity. More complex features must be extracted from the spatial properties of the simpler feature maps. Discs with heterogeneous connectivities, densities and dynamics can generate multiple complex feature maps such that the incident image can be processed into an array of independent TPs the combination of which are unique for every object. Some examples include introduction of cross talk or coupling between the discs' neurons to effectively produce filters of different spatial frequencies, the use of discs with different neuron densities and use of hardware implemented gabor filters [analogous to orientation sensitive hypercolumns in the visual cortex (Bressloff et al., 2002; Dahlem and Chronicle, 2004)] to create orientation sensitive feature maps (Choi et al., 2005; Shi et al., 2006; Chicca et al., 2007). Below we describe in more detail the last two examples and how they may be useful.

**Figure 7:**
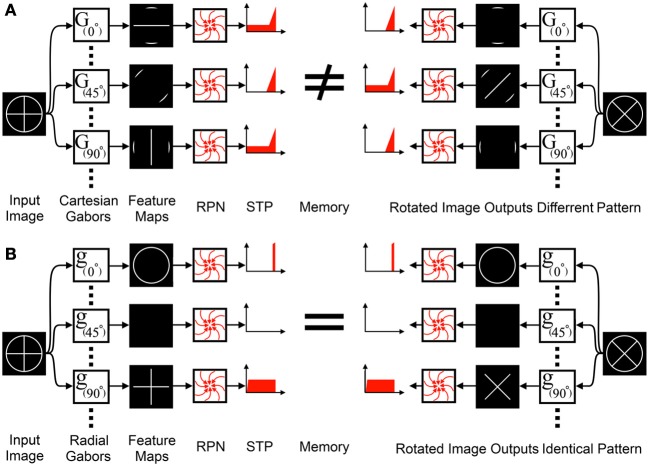
**Standard orientation sensitive feature extractors cannot precede the RPN: (A) Feature extraction via Cartesian Gabor filters, G(α), group features into feature maps based on their orientation relative to the Cartesian coordinate system**. The feature maps are input to the RPN, however, the rotational variance of the feature maps eliminates the RPN's rotation invariance. **(B)** In contrast, *radial* Gabor Filters, g(α), group features into feature maps based on their orientation relative to polar coordinates. These feature maps do not exhibit rotational variance and can interface with the RPN.

Orientation sensitive features represent a special case for the RPN. To function, the RPN and all pre-processing systems preceding it must be rotationally invariant, yet orientation sensitive feature extractors such as Gabor filters, which are a critical element of any recognition system providing salient cues that in combination are unique for different objects and operate on Cartesian coordinates. If Gabor filters that use Cartesian coordinates preceded the RPN, the resulting feature maps would be sensitive to rotation as shown in Figure 7A. A simple solution to this problem is to first transform the image into polar coordinates and then perform Cartesian Gabor filtering. This is the standard approach used in log-polar based solutions, however, this transformation and the subsequent filtering operations involves a central processor which is not biologically plausible. An alternative solution is the use of *radial* Gabor filters which group features based on their orientation relative to the disc center as shown in Figure 7 and equation (4).

(4) β=atan2(y,x)+α,θ=α+β

where,

α is the radial orientation of the radial Gabor function,

β is the angle of the position of the center of the Gabor kernel relative to the center of the disc (as shown in Figure 5),

θ is the orientation of the standard Cartesian Gabor function.

This approach has the benefit of merging the two steps into one, potentially delivering a significant speed advantage while avoiding the biologically implausible Cartesian to polar coordinate transform and, most importantly, leaves open the option of extending the RPN in a distributed manner where information about a dynamic center of attention can be used locally to generate rotationally invariant, yet information preserving feature maps.

Another potentially useful multi-disc RPN scheme would involve the use of discs with different neuron densities along their arms, which produce higher speed TPs that can reach the memory system more rapidly. These parallel high speed TPs could not only provide early information for signal normalization but can also be used to narrow the range of possible objects the image could represent such that more general categories e.g., bird vs. fish can be more rapidly determined than trout vs. carp. Recalling that patterns are represented in a temporal coding memory network as a set of signal propagation pathways, signals from the sparsely populated discs can readily be used to deactivate the vast majority of the network's pathways which do not match the early low resolution TP, thus saving most of the energy required to check a high resolution TP against every known pattern. This ensures a highly energy efficient system which rapidly narrows the number of possible object candidates with successively greater certainty.

**Figure 8:**
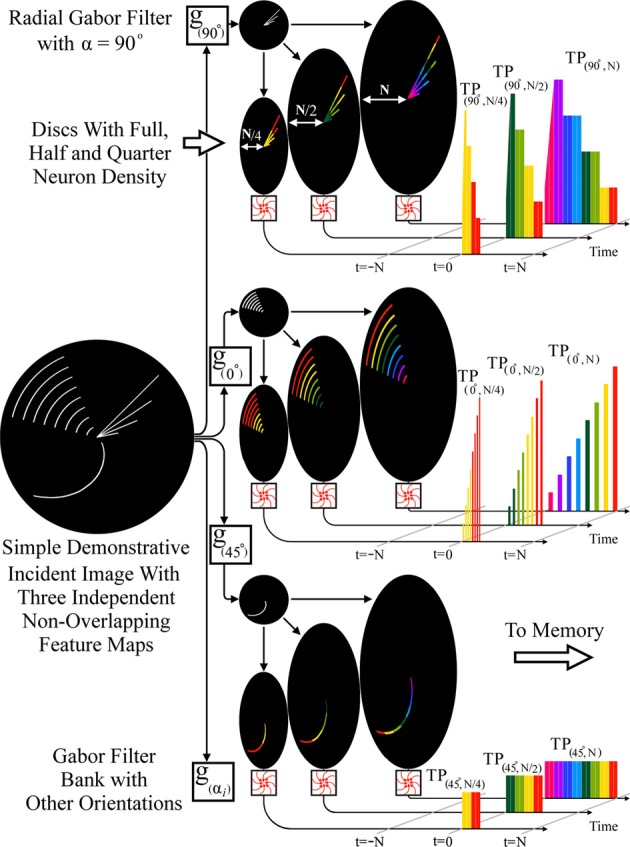
**RPN producing a spatiotemporal pattern using parallel radial Gabor filters and discs with varying neuron densities**. The incident image is processed by a filter bank of radial Gabor filters generating multiple feature maps (3 shown) each of these is then projected onto three discs with *N* (full), *N*/2 (half), and *N*/4 (quarter) density. The lower density discs simply generate an earlier low resolution version of the full TP which can be used by the memory for normalization or early categorization. The nine TPs shown illustrate the multiplicative effect of feature extractors when combined in a fan-out fashion.

As an illustration of such a fan-out feature extraction scheme, Figure 8 shows separation of an incident image via parallel, radial Gabor filters into multiple feature maps which deliver a higher dimension spatio-temporal pattern to the memory network, enabling greater selectivity. As examples, radial Gabor filters with 0, 45, and 90° orientation relative to the center are shown. Also illustrated are outputs of discs with *N* (full), *N*/2, and *N*/4 neurons on each arm, demonstrating the relative temporal order of the multi-resolution, spatio-temporal patterns generated.

The simplicity of such a parallelized, multi-scale system, the biological evidence for multi-scale visual receptive fields (Itti et al., 1998; Riesenhuber and Poggio, 1999), the presence of multispeed pathways in the visual cortex (Loxley et al., 2011) and the potential impact on energy consumption, the primary limiting factor for all biological systems, argues in favor of further investigation of such multi-scale, multispeed schemes.

## Results

To better illustrate the pertinent characteristics of the RPN we focus only on the simple one disc case without the added multi-disc extensions. Although these extensions can bring the systems performance arbitrarily close to ideal, conceptually they are repetitions of the simple case and merely make the memory system more effective by delivering more information in parallel.

### Variance of RPN output due to image transformations

**Figure 9:**
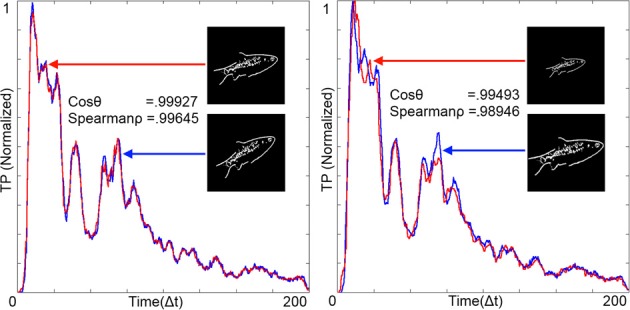
**Temporal patterns (TPs) from the RPN illustrating rotational invariance (left) and scale invariance (right)**. Images were projected on to the disc at *t* = 0 and all time scales were normalized to 200 by resampling the TPs and aligning them together. Measures of similarity between the TPs are given in the form of the Cosine Similarity (cos θ) and Spearman's Rank Correlation Coefficient (Spear'ρ). Both of these measures show high degrees of similarity between the images. Images where projected onto a disc with Φ = 200 arms and *N* = 200 neurons per arm.

The RPN is robust to rotation and scale. Figure 9 (left) shows the output TPs resulting from a 200 × 200 pixel image and its rotated equivalent. The similarity of the resulting 200 point time series was calculated via Cosine Similarity (cosθ) and Spearman's Rank Correlation Coefficient (Spear'ρ). As expected the similarity metrics were high for rotational transformation. Figure 9 (right) shows variance due to scale. The generated TPs were normalized and resampled to fill the time series vector and compared emulating the operation of the time warp invariant memory network.

**Figure 10:**
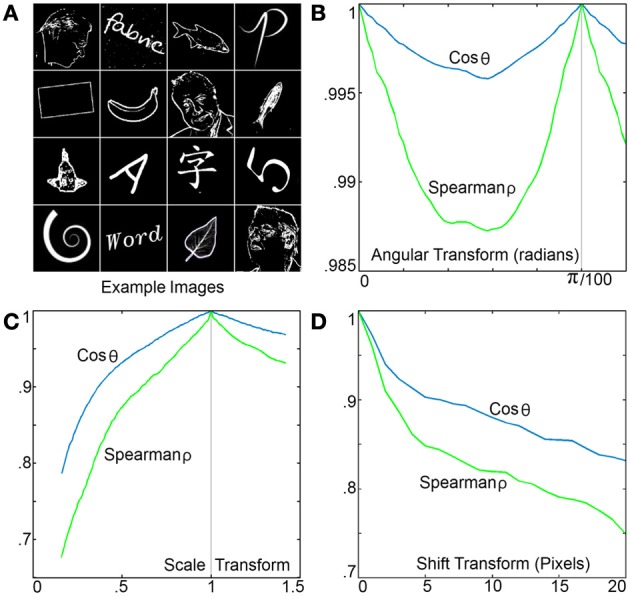
**(A)** A few of the sample images used to test the RPN's variance as a function of rotation, scale and translation. **(B)** Output TP variance as a function of image rotation, the illustrated pattern is repeated every π/100, note the small scale on the y axis. **(C)** Output TP variance as a function of image rescaling (via nearest neighbor resizing algorithm). **(D)** Output TP variance as a function of image translation demonstrating the high level of sensitivity.

To measure the RPN performance as a function of image rotation scale and shift, a mixed set of 300 different 200 by 200 pixel test images consisting of letters, numbers, words, shapes, faces, and fish were used in approximately equal numbers samples are shown in Figure 10A. All images were high-pass filtered using a difference of Gaussians kernel and processed by an RPN disc with 200 spiral arms each with 200 neurons. The similarity metrics of Spearman-ρ and cosine similarity were measured for each image across a range of rotation, translation and scale transforms with respect to the original image with the mean values shown in Figure 10B. Variance as a function of rotation is shown in the left panel, where the spiral distribution of the disc's 200 arms resulted in a high level of similarity. The pattern shown from 0 to π/100 radians is repeated as expected due to the disc's 200 arms.

Figure 10C shows variance with respect to scaling. The system shows robustness to rescaling down to low scales where the nearest neighbor image resizing operation performed to produce the downscaled images significantly reduced information content. Figure 10D shows variance due to shift or translational transform. As would be expected for a global polar transform, RPN is sensitive to non-centered images where a 10% shift of a 200 × 200 image (20 pixels) results in a drop of 0.17 and 0.25 on the cosine and Spearman similarity metrics respectively.

### Speed of operation

As a biologically inspired decentralized processor with potential use in real-world environments, the RPN's speed of operation is an important design consideration. In the context of speed the worst case for the RPN would involve two objects which fully span the RPN disc and are identical but for a distinguishing feature which is located at the disc edge (since activation at the disc edge takes the longest time to reach summing neuron), the TP from a multi-disc RPN system whose densest discs contained N neurons on each arm would be delivered to memory in:

(5) $$T_{\text{recognize}}\leq T_{\text{project}}+\Delta t_{\text{rpn}}N+T_{\text{mem}}N$$

where *T*_recognize_ is the total time needed for image recognition, *T*_project_ is the time needed for the image from the retina/sensor to be projected onto the RPN disc. In the multi-disc case this term would consist of the time required to generate the most time consuming feature maps such as hardware implemented Gabor filters resulting in *T*_project_ ≈ 240 ns (Chicca et al., 2007). Δ*t*_rpn_ is the time needed for the activation pattern to propagate one step in from the original activation point on the disc along its spiral arm. Assuming implementation via a digital relay Δ*t*_rpn_, which is in the order of nanoseconds or smaller with *N*, the number of neurons per arm on the RPN, often around 500, gives *N* × Δ*t*_rpn_ as the total amount of time for image data to be processed on the disc,. *T*_mem_ is the shortest time needed by the temporal coding memory network to process one point in the input time series and can loosely be interpreted as the temporal resolution of the memory network as its response times increase linearly with the duration of the input signal. Thus given *T*_mem_ and the length of a TP *N*, and assuming a linear relationship between the length of the input signal and time to recognition, the response time of the memory network can be estimated. With *T*_mem_ being on the order of 10 ns in current first generation hardware implementations (Wang et al., 2013), and with the same high *N*, we obtain an approximate *T*_recognize_ on the order of 5 microseconds.

Since temporal coding memory networks are able to process spatio-temporal patterns as they are being generated by RPN the Δ*t*_rpn_ term is effectively eliminated due to the temporal overlap. Which results in:

(6) $$T_{\text{recognize}}\leq T_{\text{project}}+T_{\text{mem}}N$$

Signal processing programs running on sequential von Neumann machines require computation times on the order of several milliseconds just to convert Cartesian images into log-polar images while consuming significant computational resources (Chessa et al., 2011). Hardware implemented log-polar solutions provide significantly higher speed than mapping techniques (Traver and Bernardino, 2010), however, unlike the RPN's rippling operation which generate processed TPs to memory, the log-polar foveated systems operate essentially as simple sensor grids and must interface with conventional sequential processors, introducing bottlenecks that distributed memory systems avoid. Other hardware implementations can partially bypass this bottleneck by using processor per pixel architecture or convolution networks resulting in very high speeds (Dudek, 2005; Perez-Carrasco et al., 2013) that would rival the proposed RPN solution and its extensions in speed, however, the drawback with these implementations is their lack of full view invariance.

## Discussion

### The RPN approach can be extended to three dimensions

All the features of the RPN work equally well in three dimensions, and can just as easily recognize reconstructed 3D “images.” In this context the disc is replaced by a sphere with the three dimensional image being mapped into the sphere, rippling inwards and being integrated at the center. Here the sphere does not necessarily refer to the physical shape of the network but to the conceptual structure of the connectivity, with a highly connected sphere center and radiating connectivity out to an integrating layer of neurons on the sphere surface. Given a 3D projection of an object within the sphere, skew invariant recognition could also be realized, which is among the most difficult challenges in image recognition (Pinto et al., 2008).

The reconstruction of 3D images in artificial systems is a well-developed field (Faugeras, 1993). In contrast, the underlying mechanisms performing this 3D information representation task in humans is still an area of active research (Bülthoff et al., 1995; Fang and Grossberg, 2009), where the evidence points to complex interactions between multiple mechanisms.

### Frame based visual recognition in a biological context

**Figure 11:**
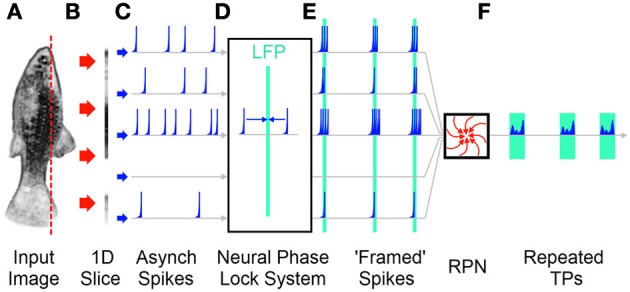
**A simplified illustration of a decentralized frame producing system without a sample and hold operation. (A)** A 2D image is projected onto the retina. **(B)** A 1D slice of the retina is illustrated. **(C)** The sensors on the retina produce spikes in an asynchronous, stochastic fashion. Each spike channel represents the path of a single “pixel” from the 2D retina through the visual system. **(D)** The asynchronous activations travel through a neural phase lock mechanism that bunches temporal patterns into frame like wave fronts of activity around Local Field Potentials (LFPs) (Martinez, 2005). **(E)** The resulting image “frames” are projected on to the RPN as described earlier. **(F)** The resulting output TPs are generated for recognition by the memory system. Note the unequal inter-frame times that would be produced due to the unclocked nature of a biological system.

As detailed earlier the RPN's conversion of 2D (or 3D) data into 1D TPs requires that the incident image be presented to the RPN disc in the form of near simultaneous wave fronts of neuronal activity, or frames such that new incoming sensory information does not corrupt processing being done on the current image. Here will follow explanation of frames. It should be noted that a frames are here defined as the coalescing of temporally distant information across a multi-channel pathway into *narrower* repeating temporal windows as illustrated in Figure 11. No statement is made about precise periodicity or precise synchrony as “framed spike” output of the neural phase lock system block in Figure 11 illustrates.

This frame-based operation of the RPN makes it more useful from a hardware implementation context, but appears to detract from its biological plausibility prompting a search for a frameless solution. Yet despite attempts to eliminate the framing requirement, to date every proposed and implemented recognition system, including those with the express goal of performing frameless event-based visual processing, such as those based on frameless vision sensors, has had to introduce some variant of a frame-based approach when attempting recognition and although the approach tends to acquire different names along the way, the final result presented to the downstream memory system is the convergence of temporally distant information by the partial slowing or arresting of the leading segments of the incoming signal (Zelnik-Manor and Irani, 2001; Lazar and Pnevmatikakis, 2011; Farabet et al., 2012; Wiesmann et al., 2012; Perez-Carrasco et al., 2013).

However, this failure may speak more to the inherent nature of the visual recognition problem than any lack of human ingenuity. Increasing evidence from neuroscience points to functional synchronicity being present in the visual cortex in the form of synchronized gamma waves where one might hypothesize an RPN or other recognition system to exist. The function of this synchronicity has been attributed to the unification of related elements in the visual field, an effect especially pronounced with attention (Meador et al., 2002; Van Rullen et al., 2007; Buschman and Miller, 2009; Fries, 2009; Gregoriou et al., 2009; Dehaene and Changeux, 2011). Furthermore the mechanisms proposed to explain such observed waves corresponds to a more distributed analog of the RPN's inhibitory neuron (Martinez, 2005), namely the inhibitory lateral and feedback connections that clump related, but spatially distant information into compact wavefronts separated by periods of inactivity. This convergence from separate fields may be pointing to the usefulness of temporal synchrony for visual inputs in the context of recognition (Seth et al., 2004).

### The shortcomings of the RPN motivates a more general solution

A significant drawback of the RPN and the one it shares with log-polar and other approaches is the need for precise centering of a salient object by an unexplained salience detection system. This system not only needs to detect objects of interest but more challengingly it must shift the image onto the RPN disc. Within the framework of centralized processing systems, the problem of shifting an image by an arbitrary value is trivial, however, in the context of distributed networks with no central control, the task is particularly challenging. A proposed solution is the use of dynamic routing systems (Olshausen et al., 1993; Postma et al., 1997) where a series of route controlling units transport the input image to a hypothetical central recognition aperture like that of the RPN disc. However, decades of neuroscientific research on the visual system has failed to find any evidence for such an aperture. Furthermore the switching speeds required to operate such control systems are far too high to be biologically achievable yet humans and animals are manifestly capable of rapid recognition of objects that are not centered on their field of view making the naïve centralized solution unlikely (van Hemmen and Sejnowski, 2005). This motivates investigation of a distributed solution to the salience detection/image centering black box. The RPN unlike previous approaches using 2D memory can easily be extended from a global image-to-TP transform to a localized operator that converts local images to local TPs such that the RPN disc can be constructed dynamically anywhere in the field of view from the gradient of the salience map enabling rapid, view invariant, multi-object recognition.

## Conclusion

In this paper we have introduced the RPN system, a simple biologically inspired view invariant transformation that is hardware implementable, and capable of converting 2D images to spatio-temporal patterns suitable for recognition by temporal coding memory networks. We described a few of the ways in which RPN can be utilized, its relationship to biological systems as well as detailing its shortcomings. With these as motivation we outlined the requirements that a more general solution would need to meet in order to be biologically plausible and useful in real world environments.

### Conflict of interest statement

The authors declare that the research was conducted in the absence of any commercial or financial relationships that could be construed as a potential conflict of interest.

## References

[B2] Adamatzky A. De Lacy Costello B. Ratcliffe N. M. (2002). Experimental reaction–diffusion pre-processor for shape recognition. Phys. Lett. A 297, 344–352 10.1016/S0375-9601(02)00289-X

[B3] Afshar S. Kavehei O. van Schaik A. Tapson J. Skafidas S. Hamilton T. J. (2012). Emergence of competitive control in a memristor-based neuromorphic circuit, in The 2012 International Joint Conference on Neural Networks (IJCNN), (Brisbane), 1–8

[B7] Avargués-Weber A. Portelli G. Benard J. Dyer A. Giurfa M. (2010). Configural processing enables discrimination and categorization of face-like stimuli in honeybees. J. Exp. Biol. 213, 593–601 10.1242/jeb.039263 20118310

[B8] Bressloff P. C. Cowan J. D. Golubitsky M. Thomas P. J. Wiener M. C. (2002). What geometric visual hallucinations tell us about the visual cortex. Neural Comput. 14, 473–491 10.1162/089976602317250861 11860679

[B8a] Brunel N. (2000). Dynamics of sparsely connected networks of excitatory and inhibitory spiking neurons. J. Comput. Neurosci. 8, 183–208 10809012 10.1023/a:1008925309027

[B9] Bülthoff H. H. Edelman S. Y. Tarr M. J. (1995). How are three-dimensional objects represented in the brain? Cereb. Cortex 5, 247–260 10.1093/cercor/5.3.247 7613080

[B10] Buschman T. J. Miller E. K. (2009). Serial, covert shifts of attention during visual search are reflected by the frontal eye fields and correlated with population oscillations. Neuron 63, 386–396 10.1016/j.neuron.2009.06.020 19679077 PMC2758537

[B11] Caley M. J. Schluter D. (2003). Predators favour mimicry in a tropical reef fish. Proc. R. Soc. Lond. Ser. B Biol. Sci. 270, 667–672 10.1098/rspb.2002.2263 PMC1691296 12713739

[B12] Carandini M. Heeger D. J. (2012). Normalization as a canonical neural computation. Nat. Rev. Neurosci. 13, 51–62 10.1038/nrn3136 22108672 PMC3273486

[B13] Cavanagh P. (1978). Size and position invariance in the visual system. Perception 7, 167–177 10.1068/p070167 652474

[B14] Chessa M. Sabatini S. P. Solari F. Tatti F. (2011). A quantitative comparison of speed and reliability for log-polar mapping techniques, in Proceedings of the 8th International Conference on Computer Vision Systems (ICVS'11), eds Crowley James L. Draper Bruce A. Monique Thonnat (Berlin, Heidelberg: Springer-Verlag), 41–50

[B14a] Chicca E. Whatley A. M. Lichtsteiner P. Dante V. Delbruck T. Del Giudice P. (2007). A multichip pulse-based neuromorphic infrastructure and its application to a model of orientation selectivity. IEEE Trans. Circuits Syst. I Regul. Pap. 54, 981–993 10.1109/TCSI.2007.893509

[B15] Choi T. Y. W. Merolla P. Arthur J. Boahen K. Shi B. E. (2005). Neuromorphic implementation of orientation hypercolumns. IEEE Trans. Circuits Syst. 52, 1049–1060 10.1109/TCSI.2005.849136

[B16] Dahlem M. A. Chronicle E. P. (2004). A computational perspective on migraine aura. Prog. Neurobiol. 74, 351–361 10.1016/j.pneurobio.2004.10.003 15649581

[B17] Dahlem M. A. Hadjikhani N. (2009). Migraine aura: retracting particle-like waves in weakly susceptible cortex. PLoS ONE 4:e5007 10.1371/journal.pone.0005007 19337363 PMC2659426

[B18] Dahlem M. A. Müller S. C. (1997). Self-induced splitting of spiral-shaped spreading depression waves in chicken retina. Exp. Brain Res. 115, 319–324 10.1007/PL00005700 9224859

[B19] Dehaene S. Changeux J.-P. (2011). Experimental and theoretical approaches to conscious processing. Neuron 70, 200–227 10.1016/j.neuron.2011.03.018 21521609

[B20] Dill M. Wolf R. Heisenberg M. (1993). Visual pattern recognition in Drosophila involves retinotopic matching. Nature 365, 751–753 10.1038/365751a0 8413652

[B21] Drazen D. Lichtsteiner P. Häfliger P. Delbrück T. Jensen A. (2011). Toward real-time particle tracking using an event-based dynamic vision sensor. Exp. Fluids 51, 1465–1469 10.1007/s00348-011-1207-y

[B22] Dudek P. (2005). A general-purpose processor-per-pixel analog SIMD vision chip, IEEE Transactions on Circuits and Systems I: Regular Papers. Vol. 52 (Kobe), 13–20 10.1109/TCSI.2004.840093

[B23] Fang L. Grossberg S. (2009). From stereogram to surface: how the brain sees the world in depth. Spat. Vis. 22, 45–82 10.1163/156856809786618484 19055887

[B24] Farabet C. Paz R. Pérez-Carrasco J. Zamarreño C. Linares-Barranco A. LeCun Y. (2012). Comparison between frame-constrained fix-pixel-value and frame-free spiking-dynamic-pixel ConvNets for visual processing. Front. Neurosci. 6:32 10.3389/fnins.2012.00032 PMC3324817 22518097

[B25] Faugeras O. (1993). Three-Dimensional Computer Vision. Cambridge, MA: The MIT Press

[B26] Fernando C. Sojakka S. (2003). Pattern recognition in a bucket, in Advances in Artificial Life SE - 63, Vol. 2801, eds Banzhaf W. Ziegler J. Christaller T. Dittrich P. Kim J. (Berlin, Heidelberg: Springer), 588–597 10.1007/978-3-540-39432-7_63

[B27] Folowosele F. O. Vogelstein R. J. Etienne-Cummings R. (2011). Towards a cortical prosthesis: implementing a spike-based hmax model of visual object recognition *in Silico*, in IEEE Journal on Emerging and Selected Topics in Circuits and Systems, Vol. 1, 516–525 10.1109/JETCAS.2012.2183409

[B28] Fries P. (2009). Neuronal gamma-band synchronization as a fundamental process in cortical computation. Annu. Rev. Neurosci. 32, 209–224 10.1146/annurev.neuro.051508.135603 19400723

[B29] Furber S. Lester D. Plana L. Garside J. Painkras E. Temple S. (2012). Overview of the spinnaker system architecture, IEEE Trans. Comput. 1 10.1109/TC.2012.142

[B30] Gao D. Vasconcelos N. (2009). Decision-theoretic saliency: computational principles, biological plausibility, and implications for neurophysiology and psychophysics. Neural Comput. 21, 239–271 10.1162/neco.2009.11-06-391 19210172

[B31] Gattass R. Nascimento-Silva S. Soares J. G. M. Lima B. Jansen A. K. (2005). Cortical visual areas in monkeys: location, topography, connections, columns, plasticity and cortical dynamics. Philos. Trans. R. Soc. B 360, 709–731 10.1098/rstb.2005.1629 PMC1569490 15937009

[B32] Gherardi F. Aquiloni L. Tricarico E. (2012). Revisiting social recognition systems in invertebrates. Anim. Cogn. 15, 745–762 10.1007/s10071-012-0513-y 22639070

[B33] Gong B. Shi Y. Sha F. Grauman K. (2012). Geodesic flow kernel for unsupervised domain adaptation, in IEEE Conference on Computer Vision and Pattern Recognition (CVPR), (Providence, RI), 2066–2073

[B34] Gregoriou G. G. Gotts S. J. Zhou H. Desimone R. (2009). High-frequency, long-range coupling between prefrontal and visual cortex during sustained attention. Science 324, 1207–1210 10.1126/science.1171402 19478185 PMC2849291

[B35] Gütig R. Sompolinsky H. (2009). Time-warp–invariant neuronal processing. PLoS Biol. 7:e1000141 10.1371/journal.pbio.1000141 19582146 PMC2701607

[B36] Hall T. S. Twigg C. M. Hasler P. Anderson D. V. (2004). Developing largescale field-programmable analog arrays, in Proceedings. 18th International Parallel and Distributed Processing Symposium. Vol. 142 (Santa Fe), 26–30 10.1109/IPDPS.2004.130312

[B37] Hamilton T. J. Tapson J. (2011). a neuromorphic cross-correlation chip, in IEEE International Symposium on Circuits and Systems (ISCAS), (Rio De Janeiro, Brazil), 865–868

[B37a] Horridge A. (2009). Generalization in visual recognition by the honeybee (Apis mellifera): A review and explanation. J. Insect Physiol. 55, 499–511 19418594 10.1016/j.jinsphys.2009.03.006

[B38] Huang X. Troy W. C. Yang Q. Ma H. Laing C. R. Schiff S. J. (2004). Spiral waves in disinhibited mammalian neocortex. J. Neurosci. 24, 9897–9902 10.1523/JNEUROSCI.2705-04.2004 15525774 PMC4413915

[B40] Huerta R. Nowotny T. (2009). Fast and robust learning by reinforcement signals: explorations in the insect brain. Neural Comput. 21, 2123–2151 10.1162/neco.2009.03-08-733 19538091

[B41] Hussain S. Basu A. Wang M. Hamilton T. J. (2012). DELTRON: neuromorphic architectures for delay based learning, in 2012 IEEE Asia Pacific Conference on Circuits and Systems (APCCAS), (Kaohsiung) 304–307 10.1109/APCCAS.2012.6419032

[B42] Iftekharuddin K. M. (2011). Transformation invariant on-line target recognition. IEEE Trans. Neural Netw. 22, 906–918 10.1109/TNN.2011.2132737 21571610

[B43] Indiveri G. Linares-Barranco B. Hamilton T. J. van Schaik A. Etienne-Cummings R. Delbruck T. (2011). Neuromorphic silicon neuron circuits. Front. Neurosci. 5:73 10.3389/fnins.2011.00073 21747754 PMC3130465

[B42a] Itti L. Koch C. Niebur E. (1998). A model of saliency-based visual attention for rapid scene analysis, IEEE Transactions on Pattern Analysis and Machine Intelligence, 20, 1254–1259 10.1109/34.730558

[B44] Itti L. Koch C. (2001). Computational modeling of visual attention. Nat. Rev. Neurosci. 2, 194–203 10.1038/35058500 11256080

[B45] Izhikevich E. M. (2006). Polychronization: computation with spikes. Neural Comput. 18, 245–282 10.1162/089976606775093882 16378515

[B46] Izhikevich E. M. Hoppensteadt F. C. (2009). Polychronous wavefront computations. Int. J. Bifurcat. Chaos 19, 1733–1739 10.1142/S0218127409023809

[B47] Jaeger H. (2001). The echo state approach to analysing and training recurrent neural networks, in GMD-Report 148, German National Research Institute for Computer Science.

[B48] Jhuang H. Serre T. Wolf L. Poggio T. (2007). A biologically inspired system for action recognition, in IEEE 11th International Conference on Computer Vision, Vol. 1 (Rio de Janeiro), 8 10.1109/ICCV.2007.4408988

[B49] Koch C. Poggio T. Torre V. (1983). Nonlinear interactions in a dendritic tree: localization, timing, and role in information processing. Proc. Natl. Acad. Sci. U.S.A. 80, 2799–2802 10.1073/pnas.80.9.2799 6573680 PMC393916

[B50] Kohonen T. (1982). Self-organized formation of topologically correct feature maps. Biol. Cybern. 43, 59–69 10.1007/BF00337288

[B51] Land M. F. (1999). Motion and vision: why animals move their eyes. J. Comp. Physiol. A 185, 341–352 10.1007/s003590050393 10555268

[B52] Lazar A. A. Pnevmatikakis E. A. (2011). Video time encoding machines. IEEE Trans. Neural Netw. 22, 461–473 10.1109/TNN.2010.2103323 21296708 PMC3758754

[B53] Levy W. B. Baxter R. A. (1996). Energy efficient neural codes. Neural Comput. 8, 531–543 10.1162/neco.1996.8.3.531 8868566

[B54] Loxley P. N. Bettencourt L. M. Kenyon G. T. (2011). Ultra-fast detection of salient contours through horizontal connections in the primary visual cortex. Europhys. Lett. 93, 64001 10.1209/0295-5075/93/64001

[B55] Maass W. (2007). Liquid computing, in Computation and Logic in the Real World SE – 53, Vol. 4497, eds Cooper S. B. Löwe B. Sorbi A. (Berlin, Heidelberg: Springer), 507–516 10.1007/978-3-540-73001-9_53

[B56] Maass W. Natschläger T. Markram H. (2002). Real-time computing without stable states: a new framework for neural computation based on perturbations. Neural Comput. 14, 2531–2560 10.1162/089976602760407955 12433288

[B58] Martinez D. (2005). Oscillatory synchronization requires precise and balanced feedback inhibition in a model of the insect antennal lobe. Neural Comput. 17, 2548–2570 10.1162/089976605774320566 16212762

[B59] McDonnell M. D. Mohan A. Stricker C. Ward L. M. (2012). Input-rate modulation of gamma oscillations is sensitive to network topology, delays and short-term plasticity. Brain Res. 1434, 162–177 10.1016/j.brainres.2011.08.070 22000590

[B60] Meador K. J. Ray P. G. Echauz J. R. Loring D. W. Vachtsevanos G. J. (2002). Gamma coherence and conscious perception. Neurology 59, 847–854 10.1212/WNL.59.6.847 12297565

[B61] Meng Y. Jin Y. Yin J. (2011). Modeling activity-dependent plasticity in BCM spiking neural networks with application to human behavior recognition. IEEE Trans. Neural Netw. 22, 1952–1966 10.1109/TNN.2011.2171044 22027373

[B62] Nakamura K. Arimura K. Takase K. (2002). Shape, orientation and size recognition of normal and ambiguous faces by a rotation and size spreading associative neural network, in Proceedings of the 2002 International Joint Conference on Neural Networks (IJCNN '02), (Honolulu) 2439–2444 10.1109/IJCNN.2002.1007524

[B63] Neri P. (2012). Feature binding in zebrafish. Anim. Behav. 84, 485–493 10.1016/j.anbehav.2012.06.005

[B64] Norouzi M. Ranjbar M. Mori G. (2009). Stacks of convolutional restricted boltzmann machines for shift-invariant feature learning, in IEEE Conference on Computer Vision and Pattern Recognition (CVPR), 2735–2742

[B65] Olshausen B. A. Anderson C. H. Van Essen D. C. (1993). A neurobiological model of visual attention and invariant pattern recognition based on dynamic routing of information. J. Neurosci. 13, 4700–4719 8229193 10.1523/JNEUROSCI.13-11-04700.1993 PMC6576339

[B67] Pardo F. Dierickx B. Scheffer D. (1998). Space-variant nonorthogonal structure CMOS image sensor design. IEEE J. 33, 842–849 10.1109/4.678644

[B68] Paugam-Moisy H. Martinez R. Bengio S. (2008). Delay learning and polychronization for reservoir computing. Neurocomputing 71, 1143–1158 10.1016/j.neucom.2007.12.027

[B69] Perez-Carrasco J. A. Zhao B. Serrano C. Acha B. Serrano-Gotarredona T. Chen S. (2013). Mapping from frame-driven to frame-free event-driven vision systems by low-rate rate-coding and coincidence processing. Application to feed forward convnets. IEEE Trans. Pattern Anal. Mach. Intell. 35, 2706–2719 10.1109/TPAMI.2013.71 24051730

[B70] Pinto N. Cox D. D. DiCarlo J. J. (2008) Why is real-world visual object recognition hard? PLoS Comput. Biol. 4:e27 10.1371/journal.pcbi.0040027 18225950 PMC2211529

[B39] Postma E. O. Van Den Herik H. J. Hudson P. T. (1997). SCAN: a scalable model of attentional selection. Neural Netw. 10, 993–1015 10.1016/S0893-6080(97)00034-8 12662495

[B71] Ranhel J. (2012). Neural Assembly Computing, IEEE Trans. Neural Netw. Learn. Syst. 23, 916–927 10.1109/TNNLS.2012.2190421 24806763

[B72] Rasche C. (2007). Neuromorphic excitable maps for visual processing. IEEE Trans. Neural Netw. 18, 520–529 10.1109/TNN.2006.884679 17385636

[B73] Reitboeck H. Altmann J. (1984). A model for size-and rotation-invariant pattern processing in the visual system. Biol. Cybern. 51, 113–121 10.1007/BF00357924 6509123

[B74] Riesenhuber M. Poggio T. (1999). Hierarchical models of object recognition in cortex. Nat. Neurosci. 2, 1019–1025 10.1038/14819 10526343

[B75] Serrano-Gotarredona R. Oster M. Lichtsteiner P. Linares-Barranco A. Rea P-V. (2009). CAVIAR: a 45k neuron, 5M synapse, 12G connects/s aER hardware sensory–processing– learning–actuating system for high-speed visual object recognition and tracking. IEEE Trans. Neural Netw. 20, 1417–1438 10.1109/TNN.2009.2023653 19635693

[B76] Serre T. Kouh M. Cadieu C. Knoblich U. Kreiman G. Poggio T. (2005). A theory of object recognition: computations and circuits in the feedforward path of the ventral stream in primate visual cortex. AI Memo 2005-036, CBCL Memo 259

[B77] Seth A. K. McKinstry J. L. Edelman G. M. Krichmar J. L. (2004). Visual binding through reentrant connectivity and dynamic synchronization in a brain-based device. Cereb. Cortex 14, 1185–1199 10.1093/cercor/bhh079 15142952

[B78] Shi B. E. Tsang E. K. C. Lam S. Y. M. Meng Y. (2006). Expandable hardware for computing cortical feature maps, in Proceedings of IEEE International Symposium on Circuits and Systems, ISCAS. (Island of Kos). 10.1109/ISCAS.2006.1693407

[B80] Sivilotti M. A. (1991). Wiring considerations in analog VLSI systems, with application to field-programmable networks. Pasadena, CA: California Institute of Technology

[B82] Sountsov P. Santucci D. M. Lisman J. E. (2011). A biologically plausible transform for visual recognition that is invariant to translation, scale, and rotation. Front. Comput. Neurosci. 5:53 10.3389/fncom.2011.00053 22125522 PMC3222220

[B83] Tapson J. van Schaik A. (2013). Learning the pseudoinverse solution to network weights. Neural Netw. 45, 94–100 10.1016/j.neunet.2013.02.008 23541926

[B84] Tapson J. C. Cohen G. K. Afshar S. Stiefel K. M. Buskila Y. Wang R. M. Hamilton T. J. van Schaik A. (2013). Synthesis of neural networks for spatio-temporal spike pattern recognition and processing. Front. Neurosci. 7:153 10.3389/fnins.2013.00153 24009550 PMC3757528

[B85] Traver V. J. Bernardino A. (2010). A review of log-polar imaging for visual perception in robotics. Robot. Auton. Syst. 58, 378–398 10.1016/j.robot.2009.10.002

[B86] Tricarico E. Borrelli L. Gherardi F. Fiorito G. (2011). I know my neighbor: individual recognition in octopus vulgaris. PLoS ONE 6:e18710 10.1371/journal.pone.0018710 21533257 PMC3076440

[B87] Van der Velden J. Zheng Y. Patullo B. W. Macmillan D. L. (2008). Crayfish recognize the faces of fight opponents. PLoS ONE 3:e1695 10.1371/journal.pone.0001695 18305823 PMC2257977

[B88] van Hemmen J. L. Sejnowski T. J. (2005). 23 Problems in Systems Neuroscience. New York, NY: Oxford University Press

[B89] Van Rullen R. Thorpe S. J. (2001). Rate coding versus temporal order coding: what the retinal ganglion cells tell the visual cortex. Neural Comput. 13, 1255–1283 10.1162/08997660152002852 11387046

[B90] VanRullen R., T. Carlson Cavanagh P. (2007). The blinking spotlight of attention. Proc. Natl. Acad. Sci. U.S.A. 104, 19204–19209 10.1073/pnas.0707316104 18042716 PMC2148268

[B91] Vogelstein R. J. Mallik U. Culurciello E. Cauwenberghs G. Etienne-Cummings R. (2007). A multichip neuromorphic system for spike-based visual information processing. Neural Comput. 19, 2281–2300 10.1162/neco.2007.19.9.2281 17650061

[B92] Volman V. Levine H. Sejnowski T. J. (2010). Shunting inhibition controls the gain modulation mediated by asynchronous neurotransmitter release in early development. PLoS Comput. Biol. 6:e1000973 10.1371/journal.pcbi.1000973 21079676 PMC2973817

[B93] Wang R. Cohen G. Stiefel K. M. Hamilton T. J. Tapson J. van Schaik A. (2013). An FPGA implementation of a polychronous spiking neural network with delay adaptation. Front. Neurosci. 7:14 10.3389/fnins.2013.00014 23408739 PMC3570898

[B94] Wang R. Tapson J. Hamilton T. J. van Schaik A. (2011). An analogue VLSI implementation of polychronous spiking neural networks, in Seventh International Conference on Intelligent Sensors, Sensor Networks and Information Processing (ISSNIP), (Adelaide) 97–102 10.1109/ISSNIP.2011.6146572

[B95] Wang X.-J. (2010). Neurophysiological and computational principles of cortical rhythms in cognition. Physiol. Rev. 90, 1195–1268 10.1152/physrev.00035.2008 20664082 PMC2923921

[B96] Wiesmann G. Schraml S. Litzenberger M. Belbachir A. N. Hofstatter M. Bartolozzi C. (2012). Event-driven embodied system for feature extraction and object recognition in robotic applications, in IEEE Computer Society Conference on Computer Vision and Pattern Recognition Workshops (CVPRW), Vol. 76 (Providence), 82 10.1109/CVPRW.2012.6238898

[B97] Wills S. (2004). Computation with Spiking Neurons. Cambridge: University of Cambridge, Ph.D. Dissertation.

[B98] Wu J. Y. Huang X. Zhang C. (2008). Propagating waves of activity in the neocortex: what they are, what they do. Neuroscientist 14, 487–502 10.1177/1073858408317066 18997124 PMC2679998

[B99] Yu Y. Santos L. M. Mattiace L. A. Costa M. L. Ferreira L. C. Benabou K. (2012). Reentrant spiral waves of spreading depression cause macular degeneration in hypoglycemic chicken retina. Proc. Natl. Acad. Sci. U.S.A. 109, 2585–2589 10.1073/pnas.1121111109 22308470 PMC3289307

[B100] Zelnik-Manor L. Irani M. (2001). Event-based analysis of video, in Computer Vision and Pattern Recognition, 2001. CVPR 2001. Proceedings of the 2001 IEEE Computer Society Conference on.

